# Expression of tyrosine pathway enzymes in mice demonstrates that homogentisate 1,2‐dioxygenase deficiency in the liver is responsible for homogentisic acid‐derived ochronotic pigmentation

**DOI:** 10.1002/jmd2.12184

**Published:** 2020-11-12

**Authors:** Peter J. M. Wilson, Lakshminarayan R. Ranganath, George Bou‐Gharios, James A. Gallagher, Juliette H. Hughes

**Affiliations:** ^1^ Department of Musculoskeletal & Ageing Science, Institute of Life Course and Medical Science University of Liverpool Liverpool United Kingdom; ^2^ Liverpool Clinical Laboratories, Department of Clinical Biochemistry and Metabolic Medicine Royal Liverpool University Hospital Liverpool United Kingdom

**Keywords:** 4‐hydroxyphenylpyruvate dioxygenase, alkaptonuria, homogentisate 1,2‐dioxygenase, LacZ reporter, tyrosinase, tyrosine hydroxylase

## Abstract

Alkaptonuria (AKU) is caused by homogentisate 1,2‐dioxygenase (HGD) deficiency. This study aimed to determine if HGD and other enzymes related to tyrosine metabolism are associated with the location of ochronotic pigment. Liver, kidney, skin, bone, brain, eyes, spleen, intestine, lung, heart, cartilage, and muscle were harvested from 6 AKU BALB/c *Hgd* ^*−/−*^ (3 females, 3 males) and 4 male C57BL/6 wild type (WT) mice. *Hgd*, 4‐hydroxyphenylpyruvate dioxygenase (*4*‐*Hppd*), tyrosine hydroxylase (*Th*), and tyrosinase (*Tyr*) mRNA expression was investigated using qPCR. Adrenal gland and gonads from AKU *Hgd tm1a −/−* mice were *LacZ* stained, followed by qPCR analysis of *Hgd* mRNA. The liver had the highest expression of *Hgd*, followed by the kidney, with none detected in cartilage or brain. Low‐level *Hgd* expression was observed within developing male germ cells within the testis and epididymis in *Hgd tm1a −/−*. 4‐*Hppd* was most abundant in liver, with smaller amounts in kidney and low‐level expression in other tissues. *Th* was expressed mainly in brain and *Tyr* was found primarily in the eyes. The tissue distribution of both *Hgd* and 4‐*Hppd* suggest that ochronotic pigment in AKU mice is a consequence of enzymes within the liver, and not from enzymatic activity within ochronotic tissues. Excessive accumulation of HGA as ochronotic pigment in joints and other connective tissues originates from the circulation and therefore the extracellular fluid. The tissue distribution of both *Th* and *Tyr* suggests that these enzymes are not involved in the formation of HGA‐derived ochronotic pigment.

**Synopsis**

Homogentisic acid accumulation in connective tissues of alkaptonuria patients is a consequence of the enzymes within the liver and not local enzymatic production at affected sites.

## 1 INTRODUCTION

**FIGURE 1:**
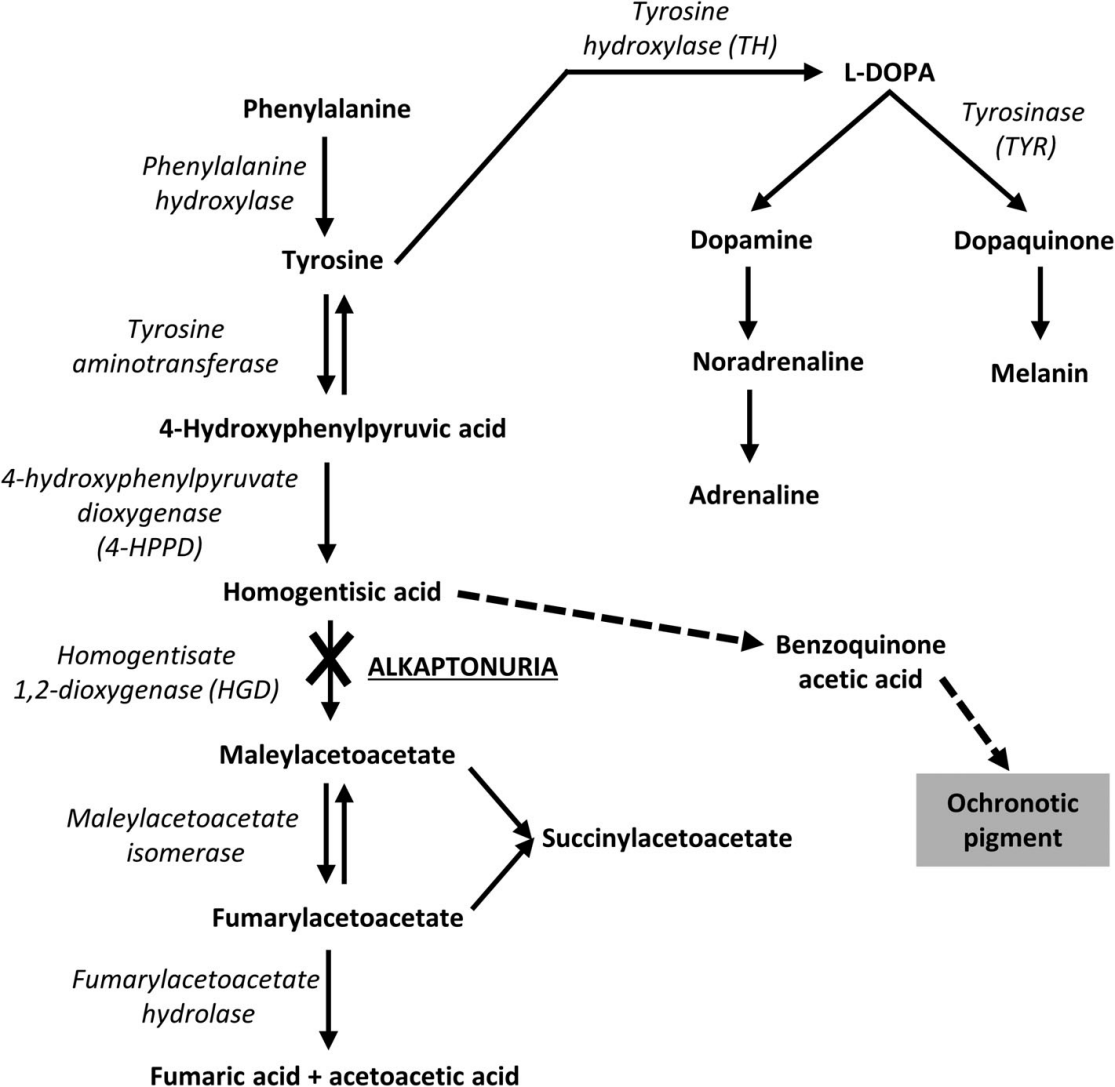
Phenylalanine/tyrosine metabolic pathway. 4‐hydroxyphenylpyruvate dioxygenase (4‐HPPD) and homogentisate 1,2‐dioxygenase (HGD) are involved in the metabolism of tyrosine to fumaric acid. Tyrosine hydroxylase (TH) is involved in the process of converting tyrosine to catecholamines and tyrosinase (TYR) in melanin production. The pathway to ochronotic pigment is shown (dashed arrows). Enzymes are in italics

Alkaptonuria (AKU; OMIM #203500) is a rare, autosomal recessive disorder of tyrosine metabolism (Figure 1) caused by the lack of homogentisate 1,2‐dioxygenase (HGD; EC 1.13.11.5).^1^ In the absence of HGD, homogentisic acid (HGA) is excreted in the urine of AKU patients, however this does not remove all excess HGA from the body. AKU is characterized by increased HGA in the plasma and therefore the body tissues, where it is thought to be oxidized and polymerized into a pigment causing ochronosis.^2^ Ochronotic pigment is deposited in the extracellular matrices and intracellular compartment of cells of connective tissues over time, especially in cartilage, leading to early onset and severe degenerative pathology in load bearing joints.^3^ In addition to joint manifestations, scleral eye pigmentation, tendon and muscle rupture, valvular heart disease and kidney stones and gallstones are features of AKU.^4^

The pathophysiological mechanism of ochronosis is currently unknown. Both HGD and 4‐hydroxyphenylpyruvate dioxygenase (4‐HPPD; EC 1.13.11.27), another enzyme in the tyrosine metabolic pathway, have been implicated in tissue localization of ochronotic pigment.^5^ It has been hypothesized that the absence of functional HGD enzyme and the expression of 4‐HPPD within localized tissue sites of ochronotic pigment deposition are its main cause, as 4‐HPPD converts 4‐hydroxyphenylpyruvate to HGA and HGD converts this to maleylacetoacetate (Figure 1). Due to the potential similarity of ochronotic pigment to melanin, both tyrosinase (TYR; EC 1.14.18.1) and tyrosine hydroxylase (TH; EC 1.14.16.2) have been implicated in ochronosis. TYR is involved in melanin production and has been hypothesized to act nonspecifically with HGA to form ochronotic pigment seen in AKU patients within specific tissue sites.^6^ TYR is located in melanocytes and is important in the production of the pigment melanin within hair, skin and eyes. TH is involved in melanin production as an intermediate enzyme, responsible for the first step of the conversion of tyrosine to L‐DOPA (Figure 1).

The aim was to determine whether production of HGA and its subsequent deposition as an ochronotic pigment (whether this be as HGA itself or as HGA‐derived pigment), is a result of local tissue enzymes in sites of ochronosis involved in tyrosine metabolism or as a result of excessive accumulation of HGA from the circulation to the extracellular fluid. Here a survey of 4 specific enzymes was conducted on tissues from albino BALB/c *Hgd* ^*−/−*^ AKU mice (*Mus musculus*).^7^ These mice have liver HGD activity of <6% of normal wild type (WT) mice,^8^ which results in increased plasma HGA at approximately 150 μmol/L and subsequent ochronosis of articular cartilage.^7^, ^9^ A point mutation (T to A, nt 1178) at the splice donor sequence of intron 12, leading to exon 12 deletion and a frameshift mutation and premature stop codon at the start of exon 13, severely truncating the protein.^10^, ^11^ To show that the tissue distribution of enzymes is not affected by albinism and the AKU defect, tissues from C57BL/6 WT mice that have a dark brown coat were also examined. Additional *LacZ* staining data is shown from a different mouse model of AKU, *Hgd tm1a −/−*, in tissues that had not been investigated previously.^12^

## 2 METHODS

All mice were housed and maintained within the University of Liverpool's Biological Services Unit (BSU) in accordance with Home Office UK guidelines. All mice were culled by cervical dislocation. Tissues were harvested for mRNA analysis from 6 BALB/c *Hgd* ^*−/−*^ (3 females, aged 56 weeks (n = 2) and 34 weeks (n = 1); 3 males, 21 weeks (n = 3)) and 4 C57BL/6 WT (male; aged 16 weeks (n = 3) and 18 weeks (n = 1)) mice (referred to as AKU and WT, respectively), which included liver, kidney, skin, bone, brain, eyes, spleen, intestine, lung, heart, cartilage, and muscle. The cartilage and eye samples were each pooled for analysis, grouped by gender. Liver, testis and epididymis tissue from *Hgd tm1a −/−* (3 males, aged 13.9 weeks) was harvested for mRNA analysis. All tissues appeared normal in color. Tissues were immediately placed into 5‐10 volumes of RNA*later* solution/tissue volume (Life Technologies) and stored overnight at 4°C. For long term storage, the RNA*later* solution was removed, tissue dried and stored at −80°C.

Approximately 35 mg of tissue was lysed using the MP FastPrep 24 with Matrix D and Matrix M (bone and cartilage) beads in 1 mL QIAZOL (Invitrogen). Seven hundred microliters of lysate was then removed for total RNA purification using the QIAGEN RNeasy mini kit, with a DNase digestion step. RNA concentration was measured using a NanoDrop Spectrophotometer (Thermo Scientific) and cDNA produced through High Capacity RNA‐to‐cDNA kit (Applied Biosystems). qPCR was performed on a Bio‐Rad CFX Connect Real‐Time PCR Detection System with SsoAdvanced Universal SYBR Green Supermix (Bio‐Rad Laboratories), with the exception of the tissues in Figure 4D, for which iQ SYBR Green Supermix was used (Bio‐Rad Laboratories).

Primers were custom designed for mouse *Hgd* 5′‐TGTCCACGGAACACCAATAA‐3′ 5′‐GCCAACTTCATCCCAGTTGT‐3′, *4‐Hppd* 5′‐ATCGCTCTCAAGACGGAAGA‐3′ 5′‐TGAGATTCTCCCGAAGCAGT‐3′, *Th* 5′‐TGTGGAGTTTGGGCTGTGTA‐3′ 5′‐AGCTTGTCCTTGGCATCACT‐3′, *Tyr* 5′‐CCTCCTGGCAGATCATTTGT‐3′ 5′‐CTGGCAAATCCTTCCAGTGT‐3′, and *18S* 5′‐GAAAATAGCCTTCGCCATCA‐3′ 5′‐AGTTCTCCAGCCCTCTTGGT‐3′ (reference gene).

Whole tissues from *Hgd tm1a −/−* mice were *LacZ* stained overnight to determine expression of *Hgd*. *Hgd tm1d (fl/fl) MxCre WT* ^12^ mice were used as a *LacZ* negative control. Adrenal gland, ovary and uterus from 13‐week *Hgd tm1a* and 6‐week *Hgd tm1d MxCre*. Testis and epididymis from 16‐week *Hgd tm1a* and 23‐week *Hgd tm1d MxCre*. Briefly, tissues were dissected, rinsed in PBS, fixed (2% paraformaldehyde, 0.2% glutaraldehyde, 0.1 M sodium phosphate buffer [pH 7.4], 5 mM EGTA and 2 mM MgCl_2_ in dH_2_0) on ice, washed 3 × 30 minutes in rinse solution (0.1 M sodium phosphate buffer [pH 7.4], 2 mM MgCl_2_, 0.1% sodium deoxycholate and 0.2% NP40 in dH_2_0) while rotating, then *LacZ* stained (1 mg/mL X‐gal (5‐bromo‐4‐chloro‐3‐indolyl‐β‐d‐galactopyranoside), 5 mM potassium ferricyanide, 5 mM potassium ferrocyanide in rinse solution) overnight in the dark while rotating. Post‐staining, tissues were fixed in formalin, paraffin embedded and sectioned.

Statistical analysis was performed using GraphPad Prism 6 statistical software. One‐way ANOVA followed by Tukey's multiple comparisons test was used to look for differences in enzyme expression between the different tissues. Error bars represent SE of the mean.

## 3 RESULTS

**FIGURE 2:**
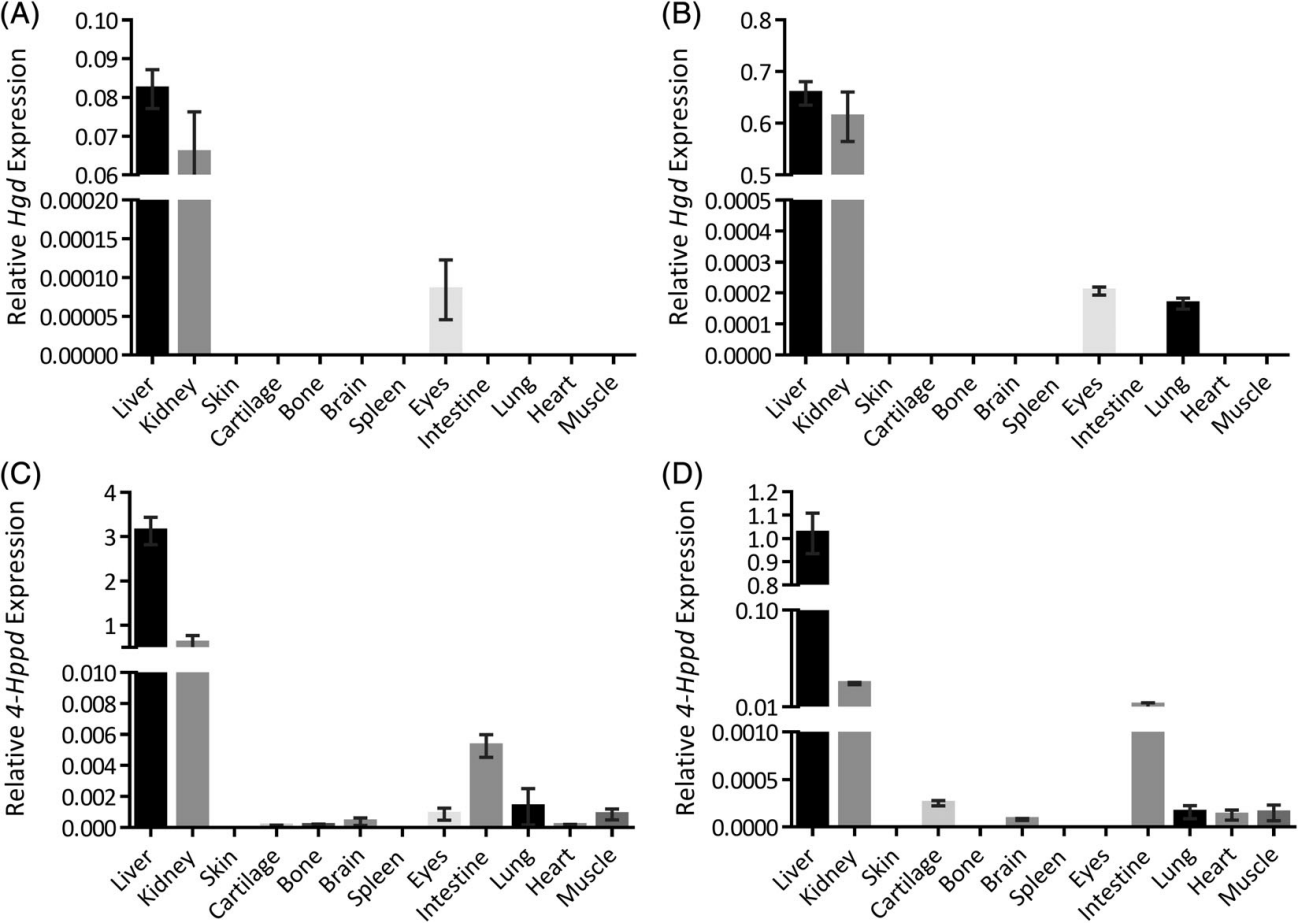
Expression of *Hgd* and *4‐Hppd*. A and B, *Hgd* and, C and D, *4‐Hppd* mRNA expression in different tissues from AKU (A and C) and WT (B and D) mice

*Hgd* mRNA was detected mainly in the liver and kidney but also observed in the eyes in both strains of mice and in the lung of the WT (Figure 2A,B). For both strains of mice, liver and kidney expression was significantly greater than all other tissues (*P* < .0001), and in AKU mice liver *Hgd* expression was significantly greater than kidney (*P* < .05). In the eyes and lung, expression was extremely low, with no expression detected in either cartilage or other tissues. *Hgd* expression was nearly 1000‐fold greater in the liver compared to the eyes in AKU mice and >3000‐fold than WT eyes.

*4‐Hppd* mRNA was primarily found in liver with smaller amounts observed in the kidney and intestine, with extremely low expression in cartilage, bone (AKU mice), brain, spleen, eyes (AKU mice; female only), lung, heart, and muscle (Figure 2C,D). *4‐Hppd* expression in liver was significantly greater than all other tissues (*P* < .0001) in both strains of mice, while kidney expression was also significantly higher than all other tissues (*P* < .01) in AKU mice, with >100‐fold expression over the intestine. For both strains of mice, liver *4‐Hppd* expression was >1500‐fold greater than all other tissues in which expression was observed (excluding the kidney and intestine), with expression in AKU mouse cartilage >15 000‐fold less.

**FIGURE 3:**
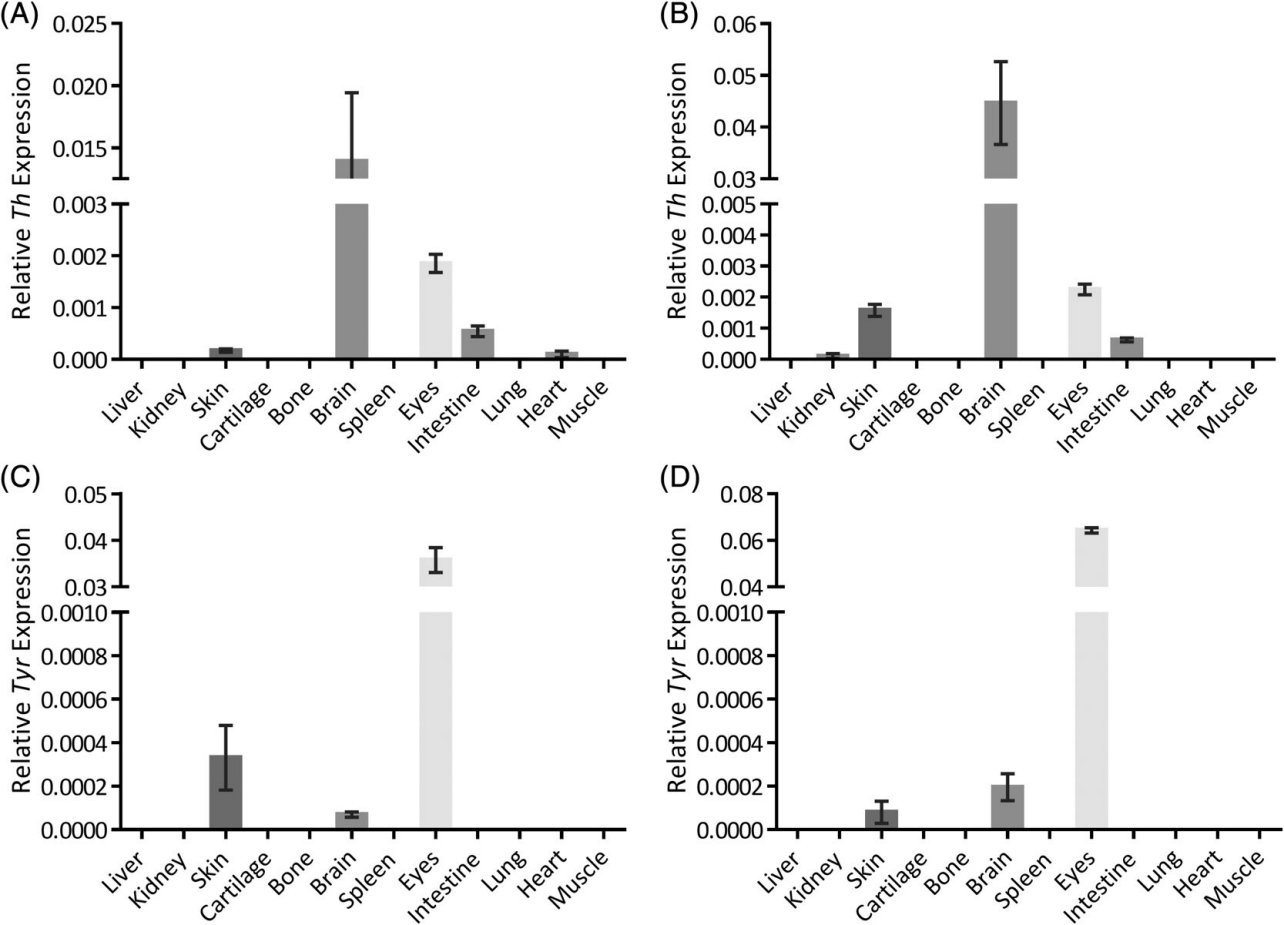
Expression of *Th* and *Tyr*. A and B, *Th* and, C and D, *Tyr* mRNA expression in different tissues from AKU (A and C) and WT (B and D) mice

*Th* mRNA was found primarily in the brain, with smaller amounts observed in the eyes, skin, intestine, heart (AKU mice) and kidney (WT mice) (Figure 3A,B). In both strains of mice, brain *Th* expression was significantly greater than other tissues (*P* < .0001). No expression was detected in the cartilage or other tissues examined. *Tyr* mRNA was mainly found in the eyes, significantly higher than other tissues (*P* < .0001), with small amounts observed in the skin and brain, and no expression in cartilage or any other tissue in both strains of mice (Figure 3C,D).

**FIGURE 4:**
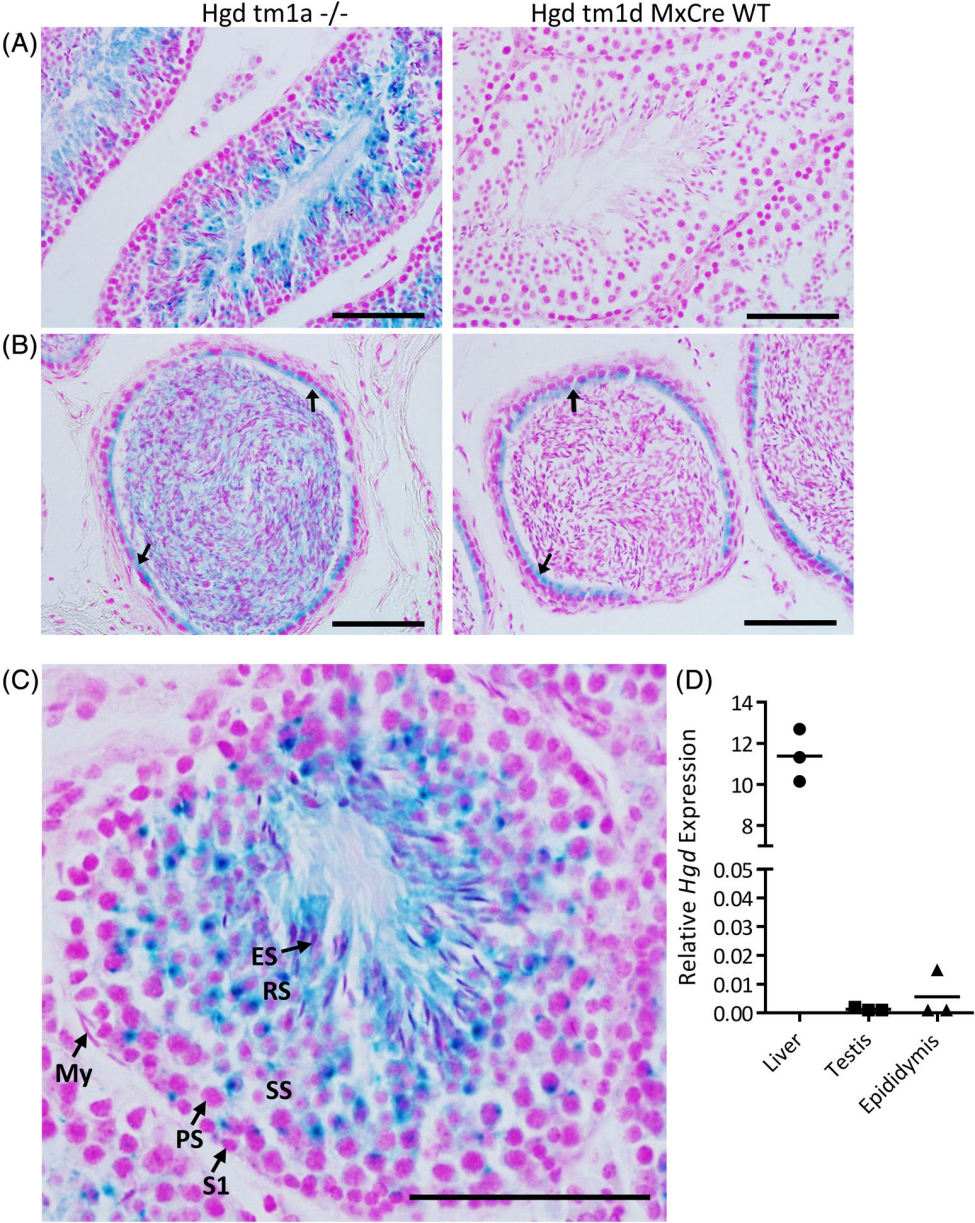
Expression of *Hgd* within developing male germ cells within the testis and epididymis. The testis, A, and epididymis, B, from *Hgd tm1a −/−* and *Hgd tm1d MxCre WT* (*LacZ* negative control) were *LacZ* stained. Arrows in (B) represent false‐positive *LacZ* staining. The cell types within the wall of the seminiferous tubules within the testis are identified in (C). *Hgd* mRNA expression within the liver, testis and epididymis of *Hgd tm1a −/−* mice is shown in (D). ES, elongated spermatozoa; My, myoid cells; PS, primary spermatocytes; RS, round spermatids; S1, spermatogonia; SS, secondary spermatocytes. Nuclear fast red counterstain in (A–C). Scale bar = 50 μm

Ex vivo staining of tissues from *Hgd tm1a −/−* which has a *LacZ* reporter gene situated within the fifth *Hgd* intron previously demonstrated *Hgd* expression in the liver and kidney proximal convoluted tubule cells, with no expression in brain, heart, lung, muscle, spleen, intestine, skin, bone, cartilage, eye, and prostate.^12^ Here, *LacZ* staining of adrenal gland, ovary and uterus from *Hgd tm1a −/−* was negative, confirmed with histological sectioning (data not shown), indicating that *Hgd* is not expressed in these tissues. *LacZ* staining of testis and epididymis however showed positive blue *LacZ* staining in *Hgd tm1a −/−* (Figure 4A,B). The staining at the luminal edge of the epididymal tubules (indicated by arrows, Figure 4B) is false‐positive, as it was also observed in the *LacZ* negative control (*Hgd tm1d (fl/fl) MxCre WT*), and therefore does not represent *Hgd* expression. There was however positive staining observed within the epididymal tubule lumen in *Hgd tm1a −/−*, that was not observed in the control. The positive staining observed in the testis appears to be localized to the cytoplasm of developing male germ cells that migrate through the seminiferous tubules, specifically within secondary spermatocytes, round spermatids and elongated spermatozoa (Figure 4C). The diffuse punctate dots of staining near the basal edge of the seminiferous tubules may be due to cytoplasmic residual bodies that are shed from elongating spermatozoa, which are then phagocytosed by the somatic Sertoli cells. The staining within the epididymis is therefore likely to be within the cytoplasm of maturing spermatozoa. *Hgd* mRNA expression (Figure 4D) in the liver of *Hgd tm1a −/−* mice was approximately 7600‐fold and 2000‐fold greater than in the testis and epididymis, respectively.

## 4 DISCUSSION

The purpose of this study was to identify whether enzymes involved in tyrosine metabolism are responsible for local production of pigment at specific tissue sites. Additionally, identifying the tissues where HGA is produced and metabolized may help target future treatments of AKU. This becomes more problematic if these enzymes are identified in tissues such as cartilage and brain, which has been previously suggested for HGD.^5^, ^13^ Articular cartilage is avascular, and due to the molecular composition of its extracellular matrix, mainly collagens and proteoglycans, it is a dense, highly anionic tissue, making drug diffusion slow and difficult to penetrate to the calcified cartilage, where initial pigmentation occurs,^14^ at a therapeutic concentration. A different challenge arises when attempting to target the brain; therapeutic molecules are required to pass through the blood‐brain barrier, which prevents both large molecules and molecules with net electric charge passing. Here we show the location of *Hgd* expression in mice is located in the liver and kidney, along with *4‐Hppd*, and rule out the involvement of melanin‐producing enzymes in the pathophysiology of AKU.

AKU patients typically become symptomatic around the fourth decade of life, presenting with progressive arthritic pain in weight bearing joints such as the spinal column, knees, and hips. It is common for patients to require multiple arthroplasty, with an average age of approximately 50 years for joint replacement surgery.^4^ Cartilage is the most affected tissue in AKU patients, so it would be reasonable to suggest that if enzymes involved in tyrosine metabolism are responsible for ochronotic pigment, that they may be detected in articular cartilage.

4‐HPPD and HGD, investigated here in mice, are enzymes involved in the metabolic process of breaking down tyrosine to fumaric acid and acetoacetic acid and have been previously reported in the liver and kidney.^1^, ^15^, ^16^, ^17^ It has been speculated that HGD is present but nonfunctional within AKU ochronotic tissues and is the cause of pigmentation.^5^ Significant *Hgd* mRNA expression however was not detected in sites of pigmentation such as articular cartilage here in mice, but was detected in both the liver and kidney as expected. Even though kidney *Hgd* expression is relatively high, intact kidney *Hgd* expression was unable to reduce plasma HGA in a conditional AKU mouse model after liver‐specific *Hgd* deletion, indicating that liver *Hgd* is crucial for HGA metabolism.^12^ Furthermore, a case report by Kobak et al^18^ reported an AKU patient's urine to be absent of HGA after a liver transplant, in addition to a halt in disease progression. *Hgd* has not been detected before in lung^12^, ^17^ or eyes^12^; the expression here observed was extremely low in these tissues, and it is not known if protein or active enzyme is present. There is no anticipated function for HGD within tissues such as the lung, consequently there could conceivably be a mechanism for downregulation of signal such as interference with siRNA's. Reports have suggested HGD expression in the brain^13^ as well as in chondrocytes and osteoblasts.^5^ Conflicting results are presented here, with no *Hgd* mRNA observed in the brain, bone or cartilage. Furthermore, northern blot analysis of *HGD* in human tissues also demonstrated no expression in brain (cartilage/bone not investigated).^17^ They did however observe *HGD* in the prostate, small intestine and colon.^17^ The *LacZ* reporter gene within the *Hgd tm1a −/−* mouse model previously found no *Hgd* expression within the eyes, lungs, brain, bone/cartilage, prostate and intestine.^12^ Here the *LacZ* reporter gene was used to investigate *Hgd* expression within the adrenal gland, ovary, uterus, testis and epididymis as they had not previously been investigated.^12^

*Hgd* expression was detected by *LacZ* staining within the cytoplasm of developing male germ cells within the testis and epididymis and not within the somatic cells. Within the seminiferous tubules, germ cells undergo spermatogenesis as they move from the basal surface of the Sertoli cell ending up as elongated, motile spermatozoa at the luminal edge.^19^, ^20^ During this process, cytoplasm is shed, leaving behind a cytoplasmic droplet in the cell; these are believed to have a role in energy metabolism essential for epididymal sperm maturation.^20^, ^21^ Downstream metabolites of HGA include fumaric acid and acetoacetic acid, which can both enter the TCA cycle and also be used for gluconeogenesis and ketogenesis, respectively. It could therefore be hypothesized that *Hgd* expression in maturing sperm a role in energy metabolism, with *Hgd* expression then downregulated in mature sperm—*Hgd* was not previously detected in the prostate via *LacZ* staining.^12^ Compared to the liver, very low *Hgd* expression in developing germ cells suggests that it is very unlikely to contribute toward metabolism of the circulating HGA pool in AKU, and is therefore likely to have no effect on pigmentation.

It has been speculated that 4‐HPPD is present and functional within AKU ochronotic tissues producing HGA.^5^ 4‐*Hppd* mRNA was most abundant in liver, with a smaller amount found in the kidney, with low expression detected in most other tissues. In humans, the skin is another site of ochronotic pigment deposition; the lack of 4‐*Hppd* mRNA here in skin and extremely low expression in cartilage, approximately 15 000‐fold less than liver in AKU mice, demonstrates that HGA present within these tissues is most likely due to circulating HGA produced by the liver. This finding is corroborated by Lin and Knox (1958), who demonstrated that liver 4‐HPPD activity greatly exceeded that of the kidney, with no significant expression in other tissues such as brain, lung, muscle and spleen within the rat.^15^ In 1972, Fellman et al reported 4‐HPPD activity in mammalian liver and kidney but not in heart, muscle or brain.^22^ The very low mRNA expression reported here within these tissues may not be enough for detectable protein or enzyme activity. Earlier studies have shown 4‐*Hppd* mRNA to be present in the rat brain^23^ but with no enzyme activity observed in the rat, monkey and human brain.^22^

The abundance of these enzymes in both the liver and kidney, especially the liver, would suggest these organs have the capacity to metabolize the tyrosine load of the whole organism. The expression of *Hgd* and 4‐*Hppd* here suggest that the ochronotic pigment found in AKU tissues is a consequence of the enzymes within the liver, affecting circulating metabolite levels that equilibrates to extracellular fluid surrounding all tissues. The liver appears to be the primary target organ for HGD replacement in AKU. Recently, it has been demonstrated that isotopically‐labeled HGA injected into the bloodstream of non‐AKU mice can be taken‐up and metabolized by *Hgd*‐expressing cells which is crucial for HGD‐replacement therapies to work effectively.^12^ As previously stated by La Du in 1998,^24^ replacement of HGD needs to be approached with caution, as the sequential steps in the tyrosine pathway produces highly reactive intermediate metabolites (maleylacetoacetate/fumarylacetoacetate/succinylacetoacetate; MAA/FAA/SAA) that must be rapidly metabolized to prevent severe liver and renal tissue damage as observed in hereditary tyrosinaemia type I (OMIM #276700) where these metabolites accumulate due to fumarylacetoacetate hydrolase (FAH; EC 3.7.1.2) deficiency. Restoring HGD activity outside of the liver could therefore cause tissue damage. La Du previously demonstrated in human AKU liver that FAH was expressed at a normal level,^1^ which provides confidence that restoring HGD activity in the liver would not cause further pathology.

HGA has been shown to oxidize to benzoquinone acetic acid (BQA) turning solutions and tissues black.^25^ Although ochronotic pigmentation has been associated with collagen,^26^, ^27^, ^28^ the mechanism of ochronotic pigmentation is unknown. It is thought that ochronotic pigment could be formed due to the oxidation of HGA to BQA as an intermediate step, although it is not known whether initial binding to collagenous matrix occurs as HGA, the BQA intermediate or as ochronotic pigment itself.^29^ A recent study using NMR and EPR spectroscopy suggests that formation of hydroquinones and radicals that causes collagen degradation in AKU cartilage.^30^ As the mechanism for ochronotic pigment formation in AKU is not known, the TH and TYR enzymes involved in the pathway for melanin production, a compound thought to be similar to ochronotic pigment, were examined for their possible role. *Th* was expressed mainly in the brain, involved in the production of catecholamine neurotransmitters, adrenaline, noradrenaline and dopamine, which are essential for normal brain function, control of physical movement and emotional behavior.^31^ TH has been previously identified in the retina,^32^ heart^33^ and kidney^34^ of mice, and intestine^35^ and skin^36^ of guinea pigs, due to association with the sympathetic nerves^37^ within these tissues. *Th* was observed to a lesser degree in some of the tissues studied here, but not detected in cartilage. TYR is responsible for the first step in melanin production, found in the retinal pigmented epithelium of the eye and melanocytes of skin. Expression of *Tyr* observed in the eyes and skin was therefore expected, which has been previously reported.^38^ Expression was also observed in the brain here, which has previously been under debate.^39^, ^40^ No expression was seen in other tissues, including cartilage. It is clear that from the tissue distribution observed of both *Th* and *Tyr*, that these enzymes are not involved in the formation of HGA‐derived ochronotic pigment at sites of ochronosis.

Ochronotic pigment is a result of exposure to high concentrations of HGA within extracellular fluid that is at equilibrium with the blood due to liver deficiency of *Hgd*, rather than through local production from enzymes within the affected tissues. The tissue distribution of *Hgd*, 4‐*Hppd*, *Th*, and *Tyr* illustrates that these enzymes are unlikely to be involved in the local formation/deposition of ochronotic pigment within connective tissues such as cartilage.

## CONFLICT OF INTEREST

The authors declare that they have no conflict of interest.

## AUTHOR CONTRIBUTIONS

Peter J. M. Wilson and Juliette H. Hughes carried out the experiment and analysis. Lakshminarayan R. Ranganath, George Bou‐Gharios, and James A. Gallagher contributed to the design of the project and analysis of data. All authors edited and approved the final manuscript.

## INFORMED CONSENT

This article does not contain any studies with human subjects performed by any of the authors. All institutional and national guidelines for the care and use of laboratory animals were followed.
